# Successful Treatment of Spinal Cord Drop Metastasis From a Forebrain Oligodendroglioma With Radiotherapy

**DOI:** 10.1111/vru.70114

**Published:** 2025-11-27

**Authors:** Angus Lane, Marc Pérez Soteras, Magdalena Parys, Jorge del Pozo, Laura Blackwood, Juan Carlos Serra

**Affiliations:** ^1^ The Royal (Dick) School of Veterinary Studies, Hospital for Small Animals The University of Edinburgh Edinburgh UK; ^2^ Fitzpatrick Referrals Orthopaedics and Neurology Godalming UK; ^3^ The Royal (Dick) School of Veterinary Studies Easter Bush Pathology The University of Edinburgh Edinburgh UK

**Keywords:** canine, drop metastasis, glioma, radiotherapy

## Abstract

A 7‐year‐old female neutered French Bulldog presented with left thoracic limb paresis. Twelve months earlier, the dog had been treated with 3D conformal radiotherapy for a right piriform lobe mass (suspected glioma), which had a strong partial response following treatment. Magnetic resonance imaging (MRI) revealed an intramedullary lesion of the cervical spinal cord, suspected to be drop metastasis. This lesion was treated with intensity‐modulated radiotherapy (IMRT) (10 fractions of 3.6 Gy, total 36 Gy), with a response documented on MRI, alongside resolution of clinical signs. The dog died of unrelated causes 647 days following IMRT, and the diagnosis was confirmed at post‐mortem examination.

**Abbreviations**
AAA: anisotropic analytic algorithm
CSF: cerebrospinal fluid
CT: computed tomography
CTV: clinical target volume
ECVAA: European College of Veterinary Anesthesia and Analgesia
FLAIR: Fluid‐attenuated inversion recovery
GA: general anesthesia
GFAP: glial fibrillary acidic protein
GTV: gross tumor volume
Gy: gray
IHC: immunohistochemistry
IMRT: intensity modulated radiotherapy
IV: intravenous
KV: kilovoltage
MRI: magnetic resonance imaging
MV: megavoltage
ODG: oligodendroglioma
PBT: primary brain tumor
PO: per os (orally)
PTV: planning target volume
QA: quality assurance
R(D)SVS: Royal (Dick) School of Veterinary Studies
RECIST: response evaluation criteria for solid tumor in dogs
RIM: radiation‐induced myelopathy
RT: radiotherapy
SRT: stereotactic radiotherapy
T1W: T1‐weighted
T1W+C: T1‐weighted post contrast
T2W: T2‐weighted
VMAT: volumetric modulated arc therapy

## 1 Signalment, History, and Clinical Findings

**FIGURE 1:**
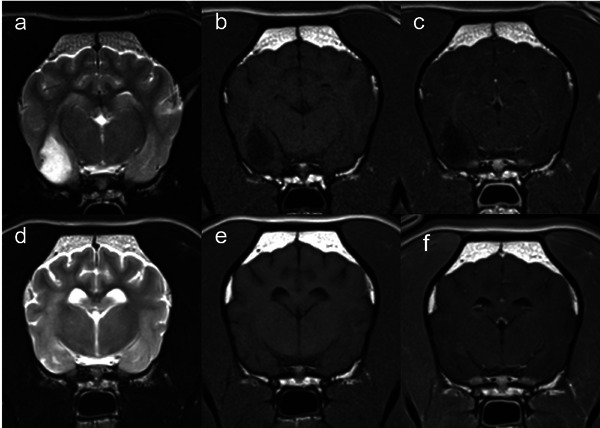
Transverse T2W (a) and (d), T1W (b) and (e), and T1W+C (c) and (f), images of the brain at the level of the thalamus. Images (a)–(c) were acquired at the time of initial presentation and showed a large, intra‐axial, relatively well‐defined mass lesion centered within the right piriform lobe. It was homogeneously T2W hyperintense compared to grey matter, T1W hypointense, and non‐contrast enhancing. The mass lesion was associated with mild mass effect, visible as mild compression and distortion of the right thalamus. Images (d)–(f) are from the MRI study 12 months post piriform mass irradiation. They document an intra‐axial, ill‐defined T2W hyperintense and T1W iso‐ to hypointense signal following the white matter tracts within the right‐sided internal capsule and corona radiata. However, the initially well‐defined, large mass lesion and associated mass effect were no longer evident. These findings were consistent with a complete response to treatment.

A 7‐year‐old female neutered French Bulldog canine (15 kg) was referred to the R(D)SVS Radiation Oncology Service for radiation therapy (RT) for a suspected glioma metastasis in the cervical spinal cord (SC). The dog had presented with left thoracic limb paresis, which developed over the course of 2 months. The dog had been treated 12 months previously for a well‐defined right piriform lobe mass (suspected glioma), with 3D conformal RT (20 fractions of 2.5 Gy, total dose of 50 Gy, prescribed to the planning target volume—PTV). The piriform lobe mass was T2‐weighted (T2W) hyperintense, T1‐weighted (T1W) hypointense, and non‐contrast enhancing mass (Figure 1).

Initial physical and neurological examination at Fitzpatrick Referrals (FR) Neurology Service was unremarkable other than for the paresis in the left thoracic limb, and the neuroanatomical localization was C1–C5. Complete blood count and serum biochemistry values were unremarkable.

Magnetic resonance imaging (MRI) of the brain and cervical SC was performed under general anesthesia (GA) using a 1.5 Tesla system (Symphony TIM, Siemens). The imaging protocol included T2W sagittal and transverse sequences; transverse T2W fluid‐attenuated inversion recovery; transverse T2W gradient echo; diffusion‐weighted imaging with corresponding apparent diffusion coefficient maps; constructive interference in steady state; and T1W transverse and sagittal sequences acquired before and after contrast administration (T1W+C). A gadolinium‐based contrast agent (gadoterate meglumine, Dotarem; 0.5 mmol/mL) was administered intravenously at a dose of 0.1 mmol/kg following acquisition of precontrast T1W images.

**FIGURE 2:**
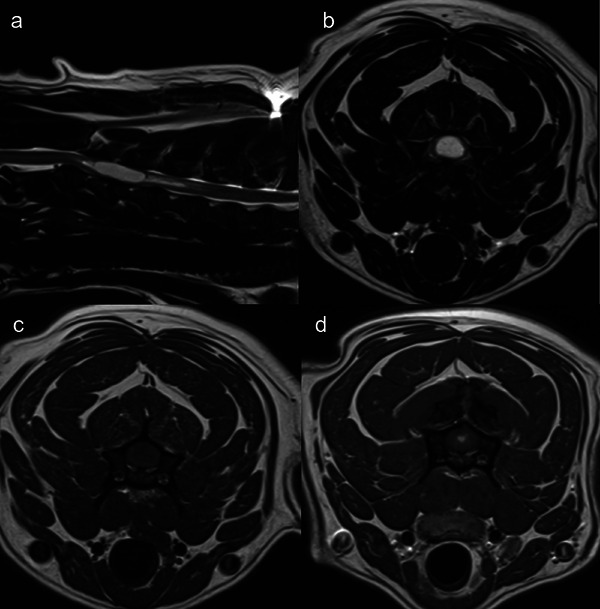
Sagittal T2W (a), transverse T2W (b), T1W (c) and T1W+C (d) images of the cervical spine. Images (b)–(d) are at the level of C3–C4 vertebral body. Images obtained prior to spinal cord lesion irradiation, revealed an elongated, fusiform, space‐occupying, intramedullary mass lesion extending from approximately mid‐C3 vertebral body almost to the end of C4 (a). The mass lesion was well‐defined, and homogeneously T2W hyperintense, T1W hypointense relative to the spinal cord, with focal, round contrast enhancement in the center of the mass, approximately at the level of C3–C4 vertebral body, (d). The maximal transverse dimension of the mass lesion was 10 mm. Additionally, there was progressive, longitudinal dilatation of the central canal cranially and caudally to the mass lesion (a). The mass caused marked mass effect, resulting in circumferential effacement of the CSF column (a and b) and occupied the central part of the spinal cord, with secondary compression of the central canal.

MRI revealed near resolution of the right piriform lobe lesion (Figure 1), and a new intramedullary, well demarcated T2W hyperintense, T1W hypointense lesion with focal, round contrast enhancement in the center of the lesion, approximately at the level of C3–C4 vertebral body (Figure 2). This was suspected to be drop metastasis from the piriform lobe mass.

The dog then presented to the R(D)SVS, where neurological examination findings were static. The dog was receiving gabapentin (11.5 mg/kg PO q12h) and levetiracetam (27.5 mg/kg PO q8h) and had been since diagnosis of the piriform lobe lesion 12 months prior. Pre‐ and post‐contrast computed tomography (CT) images of the head, SC, thorax, and abdomen were acquired using a 64‐slice helical CT (SOMATOM Definition AS, Siemens, Germany, maximum field of view 780 mm) using 2 mm axial slice thickness. Following acquisition of pre‐contrast CT, intravenous (IV) contrast was administered (2 mL/kg Omnipaque 350; GE Healthcare, Chalfont St Giles) and post‐contrast images were acquired. CT identified non‐destructive bilateral rhinitis, left sided otitis media, bilateral otitis externa, multiple vertebrae malformations, right coxofemoral degenerative joint disease, and lumbosacral degenerative disc disease. No other abnormalities were noted.

## 2 Treatment and Outcome

During CT image acquisition, the dog was positioned in sternal recumbency, with the head toward the gantry. Immobilization was achieved with a thermoplastic mould (Adapt it thermoplastic pellets, Oncology Imaging Systems, Sussex, England) over a bite plate (Health Technologies SP, Warszawa), and with a thermoplastic mask (Fibreplastic U‐frame, Oncology Imaging Systems, East Sussex, England). Crosshair marks were applied using cloth tape and permanent marker over the intersection of the CT laser at three points. Radiopaque positioning beads were placed at the 3‐laser intersection points (Suremark, Vision Line Premium Labels, V‐25, Van Arsdale, Innovative Products, Pensacola, FL, USA) and attached to the mask with adhesive. In order to contour the gross‐tumor volume (GTV) and organs at risk, T2W MRI was registered to the pre‐contrast CT. The GTV was defined as the T2W hyperintense intramedullary lesion. The clinical target volume (CTV) was defined as an extended 1 cm margin cranially and caudally inside the spinal canal, from the GTV. The CTV margin was extended three dimensionally by 5 mm, to define the PTV. Organs at risk included SC, vertebral column, trachea, and esophagus. A structure labeled “A Spine” was created, defined as [spinal_cord − (PTV + 2 mm)], to optimize dose reduction to healthy SC tissue. KV cone‐beam CT imaging was acquired daily for patient positioning verification.

Ten fractions of 3.6 Gy to a total of 36 Gy was prescribed to the PTV using a 6‐MV photon Vital Beam linear accelerator (Varian Medical Systems, Palo Alto, CA, USA). Intensity modulated radiotherapy (IMRT) was used for planning (Varian Eclipse v11; Varian Medical Systems, Palo Alto, CA, USA), and the plan had a single isocenter. Dose calculations were performed using the anisotropic analytic algorithm (AAA). The goal for radiation treatment planning was to deliver at least 95% of the prescribed dose to 95% of the PTV. Quality assurance (QA) was conducted via diode array (MapCheck3, Sun Nuclear Corporation, Melbourne, FL, USA). A minimum of 95% gamma for a 3‐mm distance to agreement and 3% absolute dose difference was defined as a passing QA score (Tables 1 and 2).

**TABLE 1:** Volume in centimeters cubed (cm^3^) of target volumes used in calculation and assessment of the radiotherapy course for spinal cord drop metastasis from oligodendroglioma.

	GTV	CTV	PTV
Drop metastasis RT—Volume of targets (cm^3^)	3.03	5.40	25.69

Abbreviations: CTV, clinical target volume; GTV, gross tumor volume; PTV, planning target volume; RT, radiotherapy.

**TABLE 2:** Radiation dose statistics for radiation target volumes in centigray (cGy) for the radiotherapy course for the spinal cord.

Dose statistic	Drop metastasis RT course—Dose to targets (cGy)		
	GTV	CTV	PTV
Min	3540	3531	3209
Max	3675	3635	3685
Mean	3572	3588	3600
Median	3590	3588	3598

Abbreviations: CTV, clinical target volume; GTV, gross tumor volume; PTV, planning target volume.

RT commenced 3 days following CT simulation. Prednisolone (0.5 mg/kg PO q24h) was prescribed at the start of treatment. Radiation fractions were administered Monday through Friday for 2 weeks on an inpatient‐basis. Each radiation fraction was delivered under GA, under the supervision of an European College of Veterinary Anesthesia and Analgesia (ECVAA)‐certified anesthesiologist. No treatment was administered on the weekend. Treatment was given once daily on all planned days successfully. An IV cannula was placed and maintained during the 12 days of hospitalization, before being removed at time of discharge.

**FIGURE 3:**
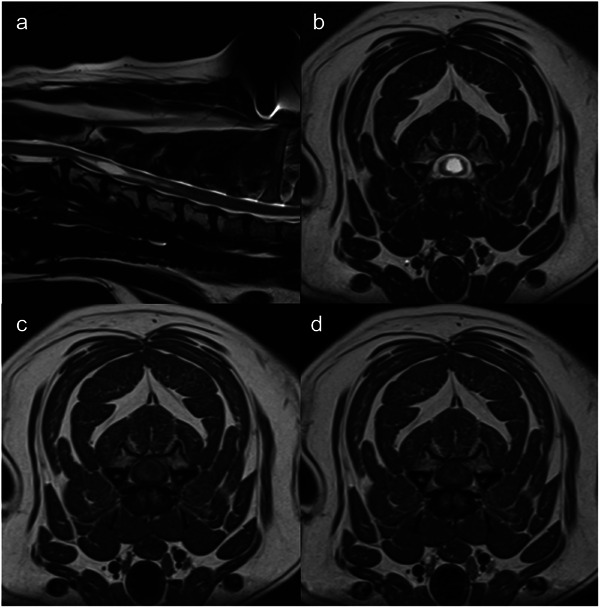
Sagittal T2W (a), transverse T2W (b), T1W (c), and T1W+C (d) images of the cervical spine. Images (b)–(d) are at the level of C3–C4 vertebral body. Images were obtained 3 months following spinal cord lesion irradiation. The fusiform, well‐defined, intramedullary mass lesion showed a reduction in size. The maximal transverse dimension of the mass lesion was reduced to 6.1 mm (39% reduction). Additionally, the CSF column exhibits normal circumferential T2W signal; visible in the sagittal (a) and transverse T2W images (b). In T1W+C images, the focal, round contrast enhancement was no longer present, with only faint, patchy contrast enhancement at the border of the mass lesion (d). These findings were consistent with a partial response to treatment.

No acute radiation toxicity was seen. Prednisolone was continued at the same dose (0.5 mg/kg PO q24h) during treatment, and for 3 weeks following, at which point the dose was tapered and continued (0.2 mg/kg PO q24h). Three months after treatment, neurological examination revealed the dog's left thoracic limb paresis had nearly resolved. MRI of the brain and cervical spine at 3 months post‐RT showed a partial response of the spinal lesion based on RECIST criteria (6.1 mm maximal transverse dimension, reduced from 10 mm pre‐treatment, a 39% reduction) (Figure 3), and static response of the primary lesion [1].

The dog presented to the primary care veterinarian with collapse, 9 months following RT for the SC lesion, and was severely anemic. She was referred to FR, where she had a thoracic and abdominal CT scan, which revealed an irregular (7 × 4 × 5 cm^3^) soft tissue attenuating and heterogeneically contrast‐enhancing mass arising within a mid‐jejunal intestinal loop. The dog was stabilized and underwent a coeliotomy, which confirmed a large, discrete mass arising from the mid‐jejunum. The mass was excised en‐bloc. Anastomosis was performed. No other abdominal abnormalities were encountered. Histopathology and immunohistochemistry (IHC) of the primary mass confirmed a leiomyosarcoma (vimentin+, smooth muscle actin+, CD117−). The dog was discharged uneventfully.

**FIGURE 4:**
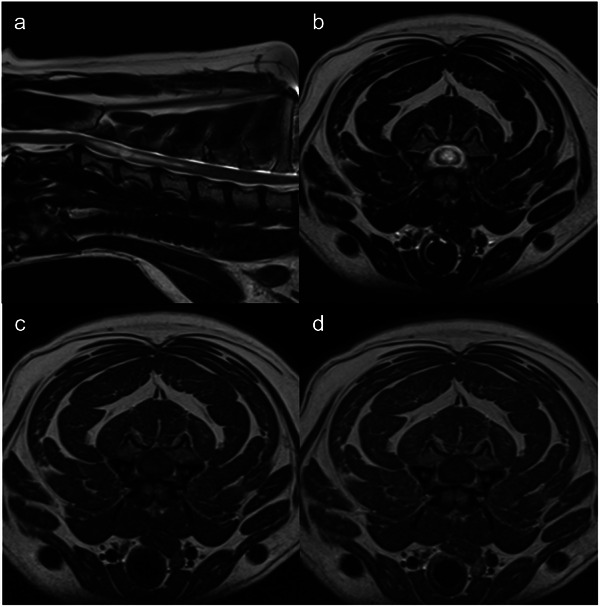
Sagittal T2W (a), transverse T2W (b), T1W (c), and T1W+C (d) images of the cervical spine. Images (b)–(d) are at the level of C3–C4 vertebral body. Images were obtained 12 months following spinal cord lesion irradiation. The initially fusiform, intramedullary mass lesion shown, was stable in maximum dimension (6.9 mm). In T1W+C images, no contrast enhancement was visible (d). These findings were consistent with an ongoing partial response to treatment.

Repeat brain and cervical spine MRI was performed at 12 months post‐RT, revealing an ongoing RECIST partial response of the spinal lesion (6.9 mm maximal transverse dimension, 13% increase when compared to MRI scan 3 months post treatment) (Figure 4), and static response of the primary lesion [1].

The dog represented to FR, 18 months post‐RT with a history of reduced exercise tolerance. Thoracic and abdominal CT scan revealed a mass, effacing the left medial and lateral liver lobes. There was no tomographic evidence of other lesions. The dog underwent a coeliotomy, the mass was excised, and it was histopathologically compatible with a metastatic lesion from the previously excised intestinal leiomyosarcoma.

The dog continued to do neurologically well for 21 months following treatment of the SC lesion, more than 2 years after treatment of the suspected piriform lobe glioma, with an unremarkable neurological examination, before euthanasia due to further abdominal metastasis from the leiomyosarcoma.

Post‐mortem examination (PME) of the central nervous system was performed. Histopathology revealed a non‐encapsulated, moderately to densely cellular proliferation of cells expanding the mesencephalic aqueduct at the level of the pons, the lateral recess of the fourth ventricle, and within the central canal of the cervical SC (C1–C4). At the SC only, the proliferating cells were infiltrating the grey matter, effacing the cord. The proliferations were composed of round cells with variably distinct cytoplasmic borders and a scant amount of eosinophilic cytoplasm, with a central, round nucleus with lacy to coarsely stippled chromatin. IHC was performed and the cells were negative for cytokeratin and vimentin, and variably positive for glial fibrillary acidic protein (GFAP) [2]. This was consistent with a diagnosis of spinal oligodendroglioma (ODG), indicating the presence of drop metastatic lesions from the previously present right piriform lobe lesion. No radiation induced histological changes were observed.

## 3 Discussion

ODG is a malignant neoplasia originating from oligodendrocytes of the neuroepithelium. ODG represents between 1% and 23% of primary brain tumors (PBTs) in the dog [3, 4]. Affected dogs usually present with intracranial signs, such as behavioral changes, obtundation, and epileptic seizures [5]. Ante‐mortem diagnosis of ODG, and of glioma more widely, is presumptive based on brain MRI [6]. Gliomas often appear poorly marginated, with variable contrast uptake [5, 6].

RT is the single most effective treatment for canine gliomas; although surgical excision and chemotherapy have been described, their efficacy is limited [4, 7, 8, 9]. It is unclear if combining RT with surgery and or chemotherapy could also potentiate tumor control [6].

With improved primary tumor control, metastases from glioma have become increasingly recognized in dogs. They typically occur months after treatment, caudal to the primary mass, following the flow of cerebrospinal fluid. Ante‐mortem diagnosis is generally imaging based, as in this case, where given the excellent response of the primary lesion to RT, irradiation of the new lesion was pursued.

An RT protocol consisting of 10 fractions of 3.6 Gy (total dose 36 Gy) was selected. This regimen aimed to balance treatment efficacy with tolerability, while limiting the number of anesthetic episodes due to the increased anesthetic risk associated with the dog's brachycephalic conformation [10].

The primary concern with SC irradiation is the risk of radiation‐induced myelopathy (RIM). The biological effective dose to the SC was similar in our protocol to a commonly used conventional fractionation protocol in humans (2 Gy × 25 fractions to a total of 50 Gy), associated with a RIM probability <5% within 5 years. The dog did not show any evidence of toxicity throughout the observed period. Although conventional fractionation has been standard, there is increasing use of more hypofractionated protocols in certain contexts, such as SC re‐irradiation, with reported low incidence of RIM [11, 12]. In veterinary medicine, inverse planning techniques such as volumetric modulated arc therapy (VMAT) have also been applied to SC neoplasia, though data are limited. Nevertheless, stereotactic radiotherapy (SRT) using 33 Gy delivered in five fractions has been used in 39 dogs—including six with SC tumors—with no reported toxicity [13].

In this case, treatment induced a partial response and clinical resolution of the neurological signs. The dog subsequently was euthanized due to an unrelated neoplasm without clinical evidence of relapse of the ODG. PME of the cervical SC confirmed the suspected diagnosis of ODG, based on a combination of histopathology and IHC (negative for vimentin and cytokeratin, variably positive for GFAP) [2].

There are a small number of case reports reporting the development of drop metastatic glioma lesions, which were not treated. Nakamoto et al. [14] describe a case of a French Bulldog diagnosed with a left piriform lobe mass, which received a hypofractionated RT protocol (7 Gy, once weekly for 7 weeks, total dose 49 Gy), alongside oral lomustine (60 mg/m^2^ every 3 weeks, from Day 30 post‐diagnosis). The dog developed seizures and was euthanized 356 days post‐diagnosis, with PME revealing drop metastases extending from the right frontal to the parietal and temporal lobes, and from the left temporal lobe to the hippocampus. Histopathological examination and IHC (oligodendrocyte transcription factor 2 positive, GFAP negative) confirmed a diagnosis of anaplastic ODG [14]. Similarly, Urso et al. [15] describe four dogs with PBTs, later confirmed to be ODG at necropsy. They were treated with VMAT (37 Gy in seven fractions or 42 Gy in 10 fractions). Temozolomide was given as a radiosensitizer (65 mg/m^2^, 6 h prior to each RT fraction, and then for 5 days monthly, for six cycles). Each dog experienced a near complete response, based on repeat MRI scan, at either 1‐ or 2‐months post‐treatment. In two cases, 4 and 9 months respectively, post‐treatment, repeat MRI scan demonstrated local recurrence as well as drop metastasis to the cervical SC. Another case developed disease progression along the entire SC, at 9 months post‐treatment. The final case presented with a gliotic scar at the primary tumor site. Findings in all dogs were confirmed at PME [15].

Vigeral et al. [16] describe treatment of a metastatic lesion. A French Bulldog received stereotactic ablative RT (three fractions of 6 Gy, on alternating weekdays) of a PBT in the rostral left lateral ventricle, the olfactory bulb, and the third ventricle—confirmed to be ODG on PME. Five weeks following RT, there was complete resolution of the mass. Drop metastatic lesions were subsequently detected on MRI, the first in the fourth ventricle, with repeat imaging revealing new lesions in C1 and C2–C3, and C4–C5. The fourth ventricle lesion was treated first with a single fraction of 10 Gy, with clinical improvement but static MR appearance. A single fraction of 6 Gy was subsequently administered to the entire nervous system, resulting in respiratory failure after recovery from GA, suspected to be related to severe cervical myelopathy [16]. This dog was included in a subsequent series of ten dogs with two or more anatomically distinct, and histologically confirmed foci of glioma, consistent with drop metastasis [17]. Seven of the 10 dogs had treatment of a primary mass and subsequently developed metastasis. Metastases were treated in two dogs: the dog reported above [16] and another that received intrathecal infusion of QUAD‐doxorubicin; however, no follow‐up was described [17].

Rancilio et al. [18] describes a 10‐year‐old boxer, which received SRT (three fractions of 8 Gy, on consecutive weekdays) for a left piriform lobe PBT, later confirmed to be an ODG on necropsy. There was a complete response of the lesion on follow‐up MRI; 6 months after RT, however, new intra‐axial lesions in the right piriform lobe, and right and left hippocampus had developed. As ODG drop metastases were suspected, another course of SRT (three fractions of 8 Gy, on consecutive weekdays) was delivered to each of the lesions. Five months after completion of the second course of SRT, a further MRI was performed, revealing new lesions in the cerebellum, brainstem and SC, which were not treated. The dog was treated palliatively prior to euthanasia, 7 months after completion of the second RT course [18]. This report did not specify the response of the drop metastases to radiotherapy.

In conclusion, to the authors’ knowledge, this is the first report to describe the successful use of RT for treatment of an ODG spinal drop metastasis. No clinically relevant early or late toxicities were present after RT, and quality of life was maintained for years, with subsequent death due to a separate neoplasia more than 2 years after treatment of the primary lesion and 21 months after treatment of the metastasis. Further research is required to explore the optimal RT protocol for treatment of spinal metastases in dogs. RT can therefore be considered for the treatment of solitary SC drop metastatic ODG lesions, when the primary tumor has been successfully managed.

## Disclosure

EVDI oral poster presentation (September 2022).

## Ethics Statement

This study complies with the Committee on Publication Ethics (COPE) guidelines and the RT protocol used in this case follows standard of care for veterinary patients. The animal owner provided written consent for the treatment provided and for the publication of data.

## Conflicts of Interest

The authors declare no conflicts of interest.

## Data Availability

Data sharing is not applicable to this article as no datasets were generated or analyzed during the current study.
